# High‐Resolution Mapping of Environmental Suitability for Reported Ebola Spillover Events in Uganda to Inform Surveillance and Preparedness

**DOI:** 10.1155/jotm/9889973

**Published:** 2026-08-16

**Authors:** George Paasi, Nancy Malaika, Sam Okware, Peter Olupot-Olupot

**Affiliations:** ^1^ Clinical Trials Department, Mbale Clinical Research Institute, Mbale, Uganda; ^2^ Department of Community and Public Health, Faculty of Health Sciences, Busitema University, Mbale, Uganda, busitema.ac.ug; ^3^ School of Medicine and Health Sciences, Soroti University, Soroti, Uganda, sun.ac.ug; ^4^ Uganda National Health Research Organisation (UNHRO), Entebbe, Uganda

**Keywords:** ebola virus disease, ecological niche modeling, MaxEnt, one health, relative suitability, Uganda

## Abstract

**Background:**

Uganda experiences recurrent Ebola disease (EBOD) outbreaks, yet national suitability maps often pool virus species or are too coarse for district‐level planning. We aimed to generate species‐resolved, 1 km relative environmental suitability surfaces for reported Ebola spillover events in Uganda; quantify the contribution of environmental, anthropogenic, and reservoir‐related predictors to modeled suitability; and translate continuous outputs into operational prioritization tiers for district surveillance and preparedness.

**Methods:**

We compiled 71 laboratory‐confirmed reported Ebola event localities in Uganda from 2000 to 2023, defined as the most likely documented locality of initial human infection where available, and paired them with 11 minimally collinear predictors. Pooled and virus‐specific MaxEnt models were tuned in ENMeval by comparing feature‐class sets across *β* = 0.5 to 3.0, ranked by AICc, and screened for acceptable omission at the 10% training‐presence threshold. Performance was estimated using fourfold spatial block cross‐validation. Pooled cloglog suitability values were thresholded at the 10% training‐presence value and stratified into four operational prioritization tiers.

**Results:**

Tuning selected LQHP with *β* = 1.5 for the pooled model. Under spatial block cross‐validation, pooled performance was Test AUC = 0.845 (0.825 to 0.862) and Test Boyce = 0.58 (0.52 to 0.63), with Test OR10 = 0.101 (0.092 to 0.109). The operational threshold was cloglog = 0.084, classifying 25.4% of Uganda as suitable within the modeling framework. Tier 1, defined as the highest‐suitability decile among suitable pixels (cloglog ≥ 0.65), covered 5.8% of land area yet contained approximately 12.6 million residents (29%). High‐suitability areas were clustered in the Albertine Rift escarpment belt, the Kampala, Wakiso, Mukono peri‐urban cluster extending along major corridors, and a northern Lake Kyoga shoreline crescent. Species‐specific outputs were interpreted as exploratory secondary analyses. SUDV broadly reproduced the pooled modeled suitability geography, EBOV showed elevated suitability along the western border corridor, and BDBV suitability was concentrated around the Albertine escarpment. Checkerboard2 sensitivity produced a small AUC increase (ΔAUC = +0.006) and high tier agreement.

**Conclusions:**

Relative environmental suitability for reported Ebola spillover localities in Uganda is concentrated in compact high‐priority belts where elevated modeled suitability overlaps with large resident populations. Tier 1 and Tier 2 areas provide a focused basis for strengthening event‐based surveillance, early detection, laboratory referral, infection prevention and control readiness, rapid response capacity, One Health field investigation, and community engagement.

## 1. Introduction

Ebola disease (EBOD) is one of the few zoonoses capable of igniting large human outbreaks from a single cross‐species event; since the first recognized episode in 1976, approximately 38,600 confirmed cases and 15,300 deaths have been documented in Africa [1]. Mounting evidence implicates fruit bats especially *Epomops franqueti*, *Hypsignathus monstrosus*, and *Myonycteris torquata* as natural reservoirs of Ebola viruses [2], while landscape drivers such as forest fragmentation, bushmeat trade, and rising human population density modulate relative environmental suitability for Ebola spillover localities [3]. Spatial risk models are indispensable for pre‐positioning surveillance and vaccine resources. MaxEnt, a presence‐background machine‐learning algorithm, is the most widely used engine for such maps because it performs robustly with sparse occurrence data and correlated predictors [4]. When MaxEnt output is transformed using the complementary log‐log (cloglog) link, the resulting values provide a relative suitability index for the environmental conditions associated with reported presence localities [5]. The available continental 5 km ecological‐niche maps [6] are limited by their coarse resolution and thus preclude actionable district‐level guidance in high‐risk countries like Uganda.

Uganda is one of only three African countries that have experienced epidemics caused by all three major human‐pathogenic Ebola viruses [7]. Since 2000, the country has recorded seven laboratory‐confirmed waves: two importation clusters of Zaire Ebola virus (EBOV) in Kasese (2019–2020) [8], a single Bundibugyo Ebola virus (BDBV) outbreak in western Uganda [9], and four Sudan Ebola virus (SUDV) outbreaks with the largest in Gulu 2000 [10]. Also, there were a solitary reemergence event in Luwero (2011) [11], another in Kibaale 2012 [12], and the Mubende outbreak in 2022 [13]. Yet, the only published national model employs 5‐km pixels and pools all species into a single surface [14]. Such coarse resolution cannot support district‐level triage, and the map predates transformative landscape shifts.

In this study, modeled suitability was interpreted as relative suitability for reported Ebola spillover localities, not as a direct measure of true zoonotic hazard or calibrated spillover probability. Reported Ebola events may reflect three linked processes: ecological hazard; human exposure opportunity; and the probability of detection, diagnosis, and reporting. MaxEnt models fitted to reported presence localities may capture signals from all three processes.

Therefore, this study aimed to generate species‐resolved, 1 km relative environmental suitability surfaces for reported Ebola spillover events in Uganda; quantify the contribution of environmental, anthropogenic, and reservoir‐related predictors to modeled suitability; and translate continuous model outputs into operational prioritization tiers for district surveillance and preparedness.

## 2. Materials and Methods

### 2.1. Study Design

This study used a retrospective spatial ecological modeling design. Laboratory‐confirmed reported Ebola spillover event localities in Uganda from 2000 to 2023 were compiled as presence records and modeled against environmental, anthropogenic, and reservoir‐related predictors using a presence‐background MaxEnt framework at 1 km resolution.

Pooled and species‐specific models were fitted to generate relative environmental suitability surfaces. Continuous outputs were thresholded and translated into operational prioritization tiers.

### 2.2. Study Area

The study was conducted in the Republic of Uganda (241,038 km^2^), a country spanning lowland savannahs (below 600 m), mid‐elevation forest and agromosaic landscapes (approximately 800 to 1400 m), and montane environments including the Rwenzori highlands (above 3000 m). All spatial analyses and maps were produced for the national extent to support district‐level preparedness planning.

### 2.3. Occurrence Data and Georeferencing

We compiled a georeferenced database of laboratory‐confirmed Ebola events in Uganda from 2000 to 2023 for SUDV, BDBV, and EBOV, drawing primarily from World Health Organization reports and Uganda Ministry of Health bulletins, supported by published case studies when available. For each event, we recorded the virus species, event date, source document, and the most specific locality information reported (Supporting Methods S1).

### 2.4. Definition of Spillover Locality

This is exactly what is in the methods and supplementary methods as the spillover locality definition. It is clearly what we defined as the spillover locality in the context of this study. Because the true location of zoonotic transmission may be uncertain, especially when exposure histories were incomplete or reported retrospectively, each occurrence record was assigned a geographic precision class reflecting the most detailed location information available. Precision classes distinguished named locality or point‐level records, parish‐level centroids, subcounty‐level centroids, district‐level centroids, and broader regional records. Records were retained for modeling only when they could be assigned at least district‐level precision, and precision classes were used to guide interpretation of fine‐scale spatial patterns. Full classification criteria are provided in Supporting Methods S1.

### 2.5. Coordinate Validation and Spatial Thinning

Occurrence coordinates were checked for plausibility and consistency with the narrative location descriptions in source documents and published case reports. To reduce the influence of clustered occurrences and to limit overfitting to near‐duplicate locations at the same modeling resolution, we applied spatial thinning using a 1 km minimum separation distance, retaining one occurrence per 1 km neighborhood, consistent with evidence that spatial filtering can reduce bias‐driven overfitting in ecological niche models [15].

### 2.6. Background Sampling and Correction for Uneven Detection Effort

Because reported EBOD events can reflect both exposure geography and spatial variation in detection and reporting opportunity, we implemented bias‐informed background sampling. We used health‐facility density as a pragmatic proxy for reporting opportunity because Ebola event detection depends partly on access to clinical assessment, alert generation, specimen referral, and diagnostic reporting pathways. A higher density of health facilities does not directly measure surveillance sensitivity, but it provides a reproducible national indicator of relative access to formal health‐system contact points. We therefore constructed a health‐facility density bias surface using Uganda Ministry of Health facility locations and supplied this raster to MaxEnt as a bias file so that background sampling better reflected the accessibility structure of reported occurrence data. This approach reduces, but does not eliminate, the possibility that low‐surveillance areas are interpreted as low suitability [16]. Full construction details are provided in Supporting Methods S2.

### 2.7. Environmental Predictors and Data Processing

We assembled 26 candidate spatial covariates representing climate, topography, human settlement and access, climate vulnerability, and reservoir‐related context. These included bioclimatic variables, elevation‐derived terrain metrics, population density, an accessibility‐based bushmeat activity proxy, ClimAfr climate‐risk indices, and a GBIF‐derived bat occurrence density proxy. All raster predictors were projected to EPSG:32636, harmonized to a common 1 km grid, aligned to the same extent and pixel origin, and clipped to the Uganda national boundary before extraction and modeling. Detailed predictor sources, preprocessing decisions, bat proxy construction, and quality‐control checks are provided in Supporting Tables S1, Supporting Methods S3 and S4.

### 2.8. Bat Occurrence Density Proxy

Bat occurrence records were filtered for coordinate completeness and quality, screened for obvious coordinate artifacts (including duplicated points and implausible locations), and processed to reduce clustered sampling before kernel smoothing. The resulting density raster was treated as a broad‐scale proxy for spatial variation in recorded bat occurrence under observation processes, not a direct measure of bat abundance. Parameter values and filtering criteria are documented in Supporting Methods S4.

### 2.9. Collinearity Screening and Final Predictor Set

To reduce redundancy and improve interpretability, we screened the 26 candidate predictors for pairwise collinearity using Pearson correlation coefficients calculated from 10,000 randomly sampled 1 km pixels across Uganda. Where predictor pairs were highly correlated, defined as |*r*| > 0.7, we retained the variable with stronger mechanistic relevance, clearer interpretability, and better suitability for operational preparedness planning. This process retained 11 minimally collinear predictors for the pooled and virus‐specific MaxEnt models (Table 1). Full screening rules and the correlation structure of the candidate predictor set are provided in Supporting Tables S2, Supporting Methods S5 and Supporting Figure S1.

**TABLE 1:** Environmental and anthropogenic predictors retained for Ebola spillover suitability modeling in Uganda.

Covariate	Description	Units	Source
Bio1	Annual mean temperature	°C	WorldClim v2.1 [17]
Bio4	Temperature seasonality (SD of monthly temperature × 100)	(SD × 100)	WorldClim v2.1 [17]
Bio12	Annual precipitation	mm	WorldClim v2.1 [17]
Bio15	Precipitation seasonality (CV of monthly precipitation)	%	WorldClim v2.1 [17]
Dem	Elevation	m	SRTM (30″) [18]
Slope	Terrain slope	Degrees	Derived from DEM [18]
Popdens	Human population density	People km^−2^	WorldPop [19]
Bushmeat	Bushmeat activity index (proxy for hunting pressure)	Unitless index	OSM and spatial proxies [20]
ClimAfr02_exposure index	Climate exposure index (degree of climatic hazards relative to baseline)	Unitless index	ClimAfr Global Climate Risk Dataset [21]
ClimAfr03_sensitivity index	Climate sensitivity index (vulnerability of systems to climatic hazards)	Unitless index	ClimAfr Global Climate Risk Dataset [21]
Bat_density	Kernel‐smoothed bat occurrence density (Chiroptera records km^−2^)	Records km^−2^	GBIF (kernel density of occurrences) [22]

### 2.10. Model Fitting, Tuning, and Evaluation

We fitted species‐resolved models (SUDV, BDBV, and EBOV) and a pooled “all‐species” model in MaxEnt v3.4.4 using the ENMeval v2.0 R package for tuning and evaluation [23]. To control model complexity and reduce overfitting, we compared five feature‐class combinations (L, LQ, H, LQH, and LQHP) across regularization multipliers from 0.5 to 3.0 in increments of 0.5. Candidate settings were ranked using corrected Akaike Information Criterion (AICc), and we required that selected configurations maintain acceptable omission performance on heldout data. Based on this tuning procedure, we selected the configuration that minimized AICc while keeping test‐fold omission below 0.10 for final model runs.

### 2.11. Spatial Cross‐Validation

To obtain performance estimates that better reflect geographic generalization, we implemented spatial cross‐validation using a block partitioning scheme. Uganda was partitioned into four latitudinal blocks, models were trained on three blocks and evaluated on the withheld block, and the process was repeated across all folds. This evaluation design reduces inflation of performance metrics caused by spatial autocorrelation and clustered occurrences and follows recommended practice for spatially structured ecological prediction [24].

### 2.12. Performance Metrics

We quantified model skill using threshold‐independent and threshold‐dependent metrics. Threshold‐independent metrics included mean area under the receiver operating characteristic curve (AUC) and the continuous Boyce index, a presence‐only evaluator designed to assess whether predictions rank observed presences toward higher suitability more than expected at random. Threshold‐dependent evaluation used omission rate at an operational threshold defined as the 10% training‐presence threshold. We report fold‐wise and summary performance statistics, prioritizing heldout fold performance for interpretation. AUC follows the evaluation framework described by Elith et al. 2011 [5], whereas the Boyce index and omission‐rate protocol follow the habitat‐suitability assessment developed by Hirzel et al. 2006 [25].

### 2.13. Variable Importance and Response Curves

To evaluate predictor contributions, we extracted MaxEnt percent contribution and permutation importance and conducted jackknife tests of training gain. We generated marginal response curves by varying one predictor at a time while holding all others at their mean to identify ranges associated with higher predicted suitability and to support mechanistic interpretation.

### 2.14. Suitability Thresholds and Risk Stratification

We applied the 10% training‐presence threshold to define an operational suitable‐area mask. We then stratified pixels within this mask into relative prioritization tiers for surveillance and preparedness planning using percentile cut points computed among suitable pixels: Tier 1 (≥ 90th percentile), Tier 2 (50th–90th percentile), Tier 3 (threshold–50th percentile), and Tier 4 (below threshold). Tiers were resampled to 100 m resolution with nearest‐neighbor interpolation for cartographic clarity, and district‐level masks were generated using the extract R package. Population counts for each tier were then extracted from the 2023 WorldPop 100 m raster and summarized nationally and across the 42 districts classified as Tier 1. These tiers represent relative modeled suitability among reported spillover localities within the MaxEnt framework.

### 2.15. Secondary Analysis: Virus‐Specific Stratification

As a secondary analysis, we stratified occurrence data by Ebola virus species and fitted separate MaxEnt models for SUDV, BDBV, and EBOV. After occurrence validation and 1 km spatial thinning, the species‐specific modeling datasets comprised 57 SUDV presence records, 7 BDBV presence records, and 7 EBOV presence records, with 10,069 background points used for each model. Species‐specific models were implemented using the same 1 km predictor stack, health‐facility‐weighted background sampling surface, tuning framework, and validation workflow applied to the pooled model. Because BDBV and EBOV were represented by very small occurrence subsets, these outputs were interpreted as exploratory secondary analyses focused on broad corridor‐level agreement rather than fine‐scale predictive performance.

### 2.16. Sensitivity Analyses

We assessed the robustness of performance and spatial patterns to the validation design by repeating evaluation under an alternative spatial partitioning scheme (checkerboard2).

All analyses were conducted in R 4.3.2 using the raster, terra, ENMeval, and ggplot2 packages, with map visualization and cartographic outputs produced in QGIS 3.34.

## 3. Results

### 3.1. Occurrence Locality Precision and Uncertainty

Occurrence localities varied in geographic precision. Records based on documented index‐case exposure localities or named settlements were considered to be higher precision, whereas records based only on administrative information were represented by centroids of the lowest reported administrative unit. This uncertainty is important in interpreting fine‐scale suitability patterns, particularly where occurrence locations were assigned at subcounty or district level rather than to a named exposure locality. The full occurrence inventory and precision categories are provided in Supporting Table S3.

### 3.2. Model Performance and Operational Threshold Selection

#### 3.2.1. Pooled Model Discrimination and Fit Diagnostics

**FIGURE 1:**
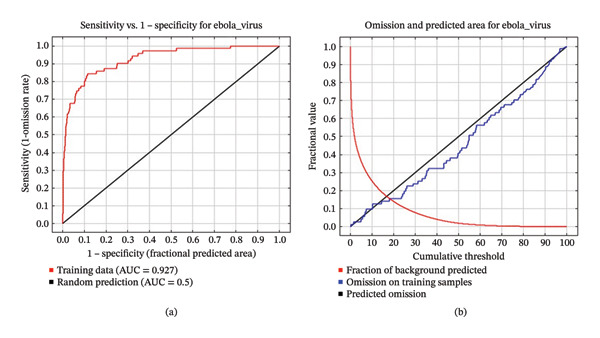
Model performance and operational threshold selection for the pooled Ebola suitability model. (a) Receiver operating characteristic (ROC) curve showing training discrimination between outbreak and background sites (training AUC = 0.927). (b) Omission predicted area curve used to select the operational 10% training‐presence threshold (cloglog = 0.084).

The pooled Ebola suitability model showed strong discrimination between reported spillover localities and the background environment. The receiver operating characteristic (ROC) curve increased steeply toward the upper‐left corner (Figure 1a), and the training AUC was 0.927, indicating that the model consistently ranked observed outbreak localities as more suitable than randomly sampled background locations.

#### 3.2.2. Threshold Justification and Predicted Suitable Area

To define an operational suitable‐area mask for downstream mapping products, we used the 10% training‐presence threshold. The omission–predicted area relationship (Figure 1b) indicates that this rule corresponds to a cloglog suitability value of 0.084. At this cutoff, the model omitted 9.9% of training localities while classifying 25.4% of Uganda’s land area as suitable. This binary suitable‐area mask was used as the basis for all subsequent tier stratification reported in later sections.

### 3.3. Model Fitting, Tuning, and Performance Metrics

For the pooled all‐species model, ENMeval tuning selected an LQHP feature set with *β* = 1.5 as the final configuration. Under fourfold spatial block cross‐validation, heldout discrimination remained strong, with mean Test AUC of 0.845 and mean Test Boyce index of 0.58. Threshold‐dependent evaluation at the 10% training‐presence threshold showed mean Test OR10 of 0.101, closely matching the intended omission level. Applying the operational threshold produced a suitable‐area estimate of approximately 25.4% of Uganda’s land area. Detailed fold‐wise performance statistics and tuning outputs are provided in Supporting Tables S4 and S5.

### 3.4. Spatial Distribution of Predicted Suitability and the Operational Suitable Mask

**FIGURE 2:**
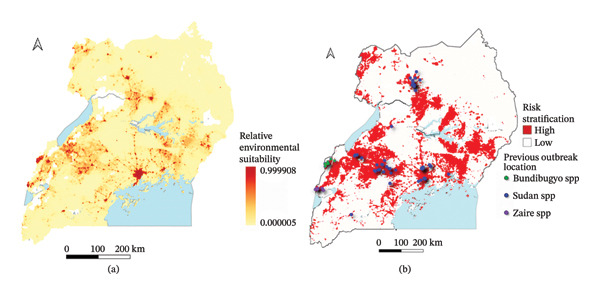
Pooled relative environmental suitability surface and operational suitable‐area mask for reported Ebola spillover localities in Uganda. (a) Continuous MaxEnt cloglog suitability values from the pooled model. Warmer colors indicate higher relative environmental suitability within the modeling framework. (b) Operational suitable‐area mask derived using the 10% training‐presence threshold. District boundaries and major water bodies are shown for orientation.

The continuous cloglog suitability surface from the pooled model revealed coherent national‐scale structure rather than isolated high‐tier suitability areas (Figure 2a). Predicted suitability was concentrated along a central corridor running through the Lake Victoria basin and extending into the central‐west, and along a western belt that tracks the Albertine Rift and adjacent escarpment zone. Within these broad corridors, suitability formed contiguous patches that align with the corridor geography, including central districts in the Kampala metropolitan region and the central‐west, and western border districts along the Rift‐adjacent belt. Outside these corridors, suitability was generally lower and more fragmented, with only localized pockets of moderate suitability in parts of the north and east.

Applying the operational threshold (10% training presence; cloglog = 0.084) converts the surface into an operational suitable mask covering approximately 25.4% of Uganda’s land area (Figure 2b). Within this suitable mask, Tier 1 covers approximately 13,980 km^2^ (5.8% of national land area) and contains about 12.6 million people. Notably, that sliver of territory contains about 12.6 million people (29% of the 2023 population) and captures all previous laboratory‐confirmed Ugandan Ebola outbreaks’ (2000–2023) localities within an 8 km buffer, validating the spatial focus of the all‐species model. Suitable pixels were not evenly distributed across the country; they were clustered strongly within the same two dominant geographies identified in the continuous surface. The largest contiguous suitable areas occurred within the central corridor spanning the Lake Victoria basin and central districts, and within the western belt near the Albertine Rift, including the Rift‐adjacent western border zone. Smaller suitable patches were present in parts of the north and east, but these were less extensive than the central and western clusters.

### 3.5. Risk Stratification of Relative Environmental Suitability for Ebola Spillover Events

Risk stratification concentrated the highest modeled suitability into a relatively small geographic footprint with substantial population exposure. Tier 1, defined as the highest‐suitability decile among suitable pixels, covered 5.8% of Uganda’s land area but included approximately 12.6 million residents, representing 29% of the 2023 population. Tier 2 expanded the prioritization envelope to 12.5% of land area and included approximately 15.8 million residents. Together, Tier 1 and Tier 2 accounted for 18.3% of national land area but approximately 65% of the population, highlighting a concentrated set of areas where intensified surveillance and preparedness may be most operationally relevant. Full tier‐specific area, population, and district summaries are provided in Table 2.

**TABLE 2:** Area and population exposure estimates for suitability tiers from the pooled model.

Tier	Cloglog range	Land area (% of Uganda)	Population (approx.)	No. of districts
Tier 1 (High)	≥ 90th percentile (cloglog ≥ 0.65)	5.8	∼12.6 million (29%)	42
Tier 2 (Medium)	50th–90th percentile (cloglog 0.28–0.65)	12.5	∼15.8 million (36%)	52
Tier 3 (Low)	Threshold–50th percentile (within suitable pixels)	27.1	∼10.2 million (23%)	30
Tier 4 (Very low)	Below threshold (nonsuitable)	54.6	∼6.4 million (12%)	22
Total	—	100.0	∼45.0 million (100%)	—

**FIGURE 3:**
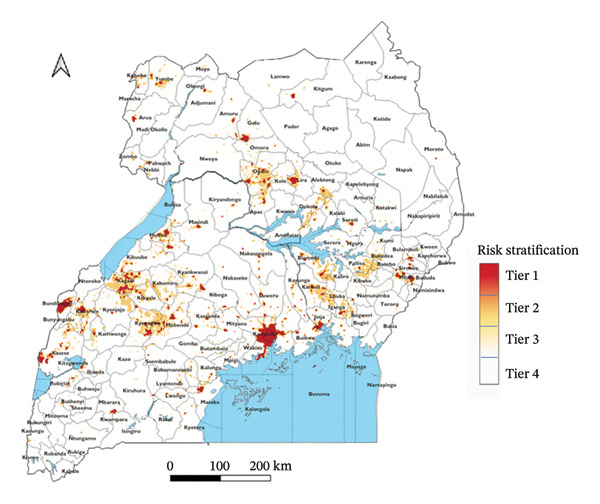
Tiered operational prioritization map derived from the pooled relative suitability surface. Tier 1 represents the highest‐suitability decile among pixels above the operational threshold. Tier 2 represents the 50th to 90th percentile among suitable pixels. Tier 3 represents pixels between the operational threshold and the 50th percentile. Tier 4 represents pixels below the operational threshold. These tiers are relative prioritization classes for surveillance and preparedness planning.

Figure 3 shows a four‐tier Ebola relative environmental suitability map for Uganda. Tier 1 is restricted around the Central region of Kampala–Wakiso–Mukono metropolitan core and extends along two secondary corridors: the Albertine rift chain running through Kabarole, Bunyangabu, Hoima, and Bundibugyo, and a central belt that links Masindi through Mubende to Luweero/Nakaseke‐Nakasongola. Tier 2 widens the envelope into the surrounding peri‐urban and market‐town catchments Jinja and Iganga on the Lake Victoria littoral, Mbale and Sironko on the Mt Elgon slopes, the mid‐northern growth poles of Gulu and Arua, and the Lake Kyoga crescent that includes Kwania, Apac, Dokolo, and Soroti. The remainder of the country falls into Tier 3; this class dominates the semi‐arid Karamoja cluster in the north‐east (Kotido, Kaabong, Moroto, Napak, Abim, and Amudat) and the high‐altitude south‐western districts flanking the Rwenzori and Kigezi highlands (Kasese, Kanungu, Rukungiri, Kabale, Kisoro, Ntungamo, Rubanda, and Bundibugyo’s high ranges).

### 3.6. Predictor Importance and Response Patterns

#### 3.6.1. Variable Contribution and Permutation Importance

After quantifying percent contribution and permutation importance for each predictor (Table 3), we assessed each variable’s unique and combined information using a jack‐knife test.

**TABLE 3:** Percent contribution, permutation importance, and response‐curve features for all 11 covariates of the all‐species MaxEnt model.

Rank	Covariate	% contribution	Permutation importance (%)	Response‐curve feature
1	Human population density (Popdens)	77.4	66.9	Logistic rise between 100 and 500 people km^−2^, plateau thereafter.
2	Bushmeat market accessibility (Bushmeat)	9.5	9.2	Monotonic increase; pure‐presence curve peaks at index ≈ 2.0 then falls.
3	Fruit‐bat roost density (Bat_density)	7.7	12.8	Hump‐shaped optimum at 0.08–0.12 roosts km^−2^; decline beyond 0.25.
4	Precipitation seasonality (Bio15)	6.9	8.9	Hump‐shaped; intermediate optimum for rainfall variability.
5	Annual mean temperature (Bio1)	3.6	10.8	Monotonic increase in suitability with temperature.
6	Climate sensitivity index (ClimAfr03)	3.1	6.5	Inverted‐U with optimum at ≈ 2.5; sharp decline > 3.1
7	Climate exposure index (ClimAfr02)	1.6	3.7	Sigmoid increase above index ≈ 2.3
8	Elevation (DEM)	0.2	0.9	Peak suitability at 800–1400 m; rapid decline above 2200 m
9	Terrain slope	0.4	0.0	Negligible marginal influence
10	Temperature seasonality (bio4)	0.1	0.4	Very weak effect on suitability
11	Annual precipitation (bio12)	0.0	0.1	Essentially no marginal effect

#### 3.6.2. Jackknife Analysis of Training Gain

**FIGURE 4:**
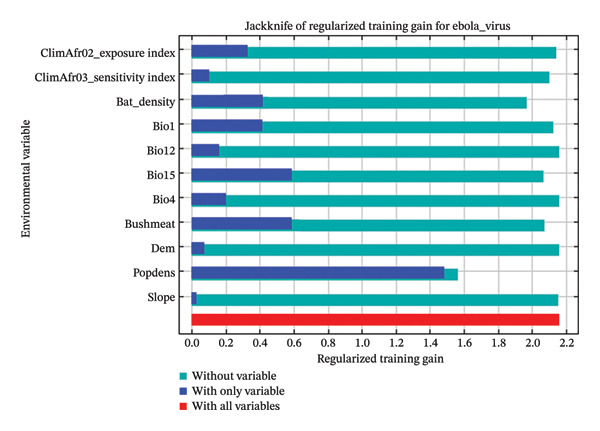
Jack‐knife analysis of variable importance for the pooled‐species MaxEnt model. Blue bars show the training gain with each variable in isolation; turquoise bars show the drop in gain when each variable is omitted from the full model.

The jack‐knife of regularized training gain (Figure 4) reveals that human population density contributes the most independent information producing the highest gain when used alone and causing the largest drop when omitted. Bushmeat accessibility and fruit‐bat roost density are the next most informative variables, each adding a unique signal beyond population density. Precipitation seasonality (Bio15) and annual mean temperature (Bio1) also provide appreciable unique gains, whereas temperature seasonality (Bio4) and annual precipitation (Bio12) contribute minimal unique information. In contrast, elevation, slope, and the ClimAfr composites each contribute comparatively little unique gain, indicating their roles as broad‐scale filters rather than primary drivers. Jack‐knife tests and permutation importance placed the 11 covariates in a clear hierarchy.

#### 3.6.3. Marginal Response Curves for the Predictors

**FIGURE 5:**
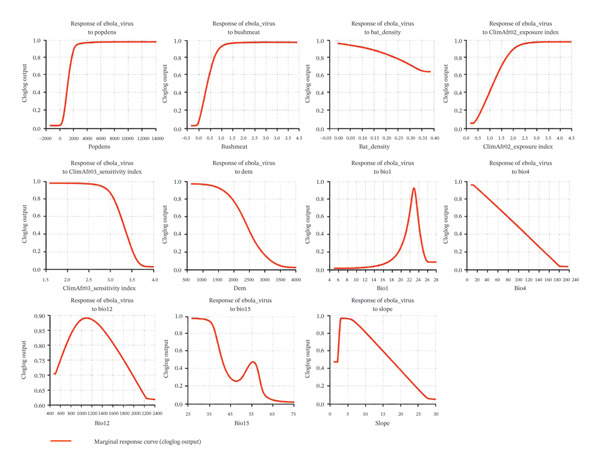
Marginal response curves for the all‐species Ebola MaxEnt model. Panels show fitted MaxEnt cloglog suitability values across covariate gradients. Cloglog values are interpreted as relative environmental suitability within the modeling framework.

The marginal response curves (Figure 5) reveal each predictor’s mechanistic contribution to the all‐species modeled suitability.

Human population density showed the strongest association with modeled suitability, with low fitted suitability below approximately 100 persons km^2^, a steep increase between approximately 100‐ and 500‐persons km^2^, and a plateau thereafter. This response should be interpreted as a combined signal of human exposure opportunity, outbreak detection and reporting intensity, and possible outbreak amplification, rather than as evidence that population density is a direct ecological driver of zoonotic spillover.

Bushmeat accessibility increases almost linearly from ∼0.64 at index 0 to ∼1.0 by index 1.8; fruit‐bat roost density shows a hump‐shaped response, peaking at ∼0.08 roosts km^−2^ then declining beyond ∼0.25; annual mean temperature (Bio1) displays a unimodal thermal niche minimal suitability below ∼15°C, a peak at ∼22°C, and a decline above ∼23°C; precipitation seasonality (Bio15) exhibits a bimodal pattern high suitability at low values (< 35), a trough at ∼50, a minor secondary hump near ∼55, then near‐zero suitability at high seasonality (> 65); climate exposure (ClimAfr02) shows a sigmoid rise above ∼2.3, plateauing by ∼3.5; climate sensitivity (ClimAfr03) forms an inverted‐U, with high suitability from ∼1.3 to 2.7 and a sharp drop past ∼3.1; elevation imposes a monotonic decline maximum at 500–1000 m, near zero above 3500 m; terrain slope has negligible effect; temperature seasonality (Bio4) declines steadily from ∼0.90 at low variability (< 20) to ∼0.62 at high variability (∼220); and annual precipitation (Bio12) exhibits a unimodal response near zero below ∼600 mm, peaking at ∼1500 mm, then declining to ∼0.43 above ∼2000 mm.

### 3.7. Species‐Specific Models as Secondary Analyses

Because the number of confirmed presence records differed substantially by virus species, the species‐specific models were interpreted with different levels of confidence. The SUDV model was supported by a larger occurrence subset, whereas the BDBV and EBOV models were based on only seven confirmed presence records each. For these small‐sample models, high training AUC values should be interpreted as descriptive indicators of within‐sample discrimination only and not as evidence of strong predictive generalization. Accordingly, the BDBV and EBOV outputs are treated as exploratory secondary analyses, with interpretation restricted to broad spatial agreement and corridor‐level patterns rather than fine‐scale prediction.

### 3.8. SUDV Species‐Specific Relative Suitability Surface

The SUDV MaxEnt model was trained on 57 confirmed SUDV presence points and 10069 background pixels, converging after 500 iterations with a regularized training gain of 1.967 and a training AUC of 0.961. The omission‐predicted‐area curve identified the 10% training‐presence cloglog threshold at 0.083, which retains 90% of known SUDV localities while classifying 25.4% of Uganda’s land area as suitable (fractional predicted area = 0.254) (Supporting Figure S2.1). The continuous suitability map highlights a core high‐suitability belt (cloglog ≥ 0.70) that arcs from Bundibugyo–Ntoroko across the mid‐elevation escarpment through Kagadi, Kibaale, and Hoima, then swings east into the Kampala–Wakiso–Mukono peri‐urban zone. A secondary arm follows the northern Lake Kyoga shoreline (Apac to Kwania to Oyam), while the Karamoja semi‐arid northeast and the Kisoro–Kabale highlands remain uniformly unsuitable (< 0.10) (Supporting Figure S2.2). Applying the 0.083 threshold yields a binary high‐risk mask covering 33 of 146 districts (23%), encompassing ≈ 9900 km^2^ (4.9% of national land) and ≈ 10.8 million people (25% of Uganda’s 2023 population).

### 3.9. BDBV Species‐Specific Relative Suitability Surface

The BDBV MaxEnt model was built with 7 confirmed presence records and 10069 background points, converging after 100 iterations to a regularized training gain of 4.421 and a training AUC of 0.999 (Supporting Figure S2.3). Although the model produced a very high training AUC, this value is likely influenced by the small occurrence subset and should be treated as a descriptive within‐sample diagnostic rather than evidence of reliable predictive performance. The continuous suitability map shows higher relative suitability values (cloglog ≥ 0.70) concentrated along the Albertine rift escarpment notably Ntoroko, Bundibugyo, Kabarole–Kyenjojo–Kikuube, and Kanungu–Rukungiri, with only a small, isolated nucleus over Kampala–Wakiso. Binarizing at the 10% training‐presence cloglog threshold of 0.524 (retaining 90% of presences) reduces the suitable‐area mask to 0.3% of Uganda’s land area (fractional predicted area = 0.003) and flags 14 of 146 districts (10%) as high‐risk districts (Supporting Figure S2.4).

### 3.10. Zaire Ebolavirus Species‐Specific Relative Suitability Surface

The EBOV MaxEnt model was fit using 7 presence records and 10069 background points. It converged after 100 iterations with a regularized gain of 1.388 and a training AUC of 0.993 (Supporting Figure S2.5). As with the BDBV model, the very high training AUC should be interpreted cautiously because small presence datasets can produce inflated discrimination metrics and unstable fitted surfaces. The continuous suitability map indicates a high‐suitability corridor (cloglog ≥ 0.70) along Uganda’s western border with the Democratic Republic of the Congo, extending from the Kisoro–Kanungu highlands north through the Rwenzori foothills. Elsewhere, central and eastern Uganda remain pale (< 0.15). Thresholding at the 10% training‐presence cloglog cutoff of 0.086 produces a binary suitable‐area mask covering just 9029 km^2^ (≈ 4.5% of Uganda) that falls entirely within 14 border districts and encompasses ≈ 4.0 million people (9.1% of the 2023 population) (Supporting Figure S2.6).

### 3.11. Cross‐Model Comparison

Across species, the primary high‐suitability geography remains anchored to western and central‐west Uganda, but the spatial emphasis differs in ways that are consistent with species stratification (Table 4). The SUDV model broadly resembles the pooled model’s central corridor, including the Kampala to Hoima corridor and the Albertine‐facing belt, and adds a clearer secondary arm along the Lake Kyoga shoreline. In contrast, the BDBV model concentrates suitability tightly along the Albertine Rift escarpment with minimal inland extension, and the EBOV model concentrates suitability in a narrow western border ribbon.

**TABLE 4:** Summary of pooled and species‐specific model outputs, including number of occurrence localities, training AUC, operational threshold value, predicted suitable area proportion, and key high‐tier suitability regions.

Model	Occurrence localities (*n*)	Training AUC	Operational threshold (10% training‐presence, cloglog)	Predicted suitable area (proportion of Uganda)	Key high‐tier suitability regions (broad patterns)
Pooled (all species)	71	0.927	0.084	0.254 (25.4%)	Albertine escarpment belt (Kisoro to Kanungu north through Rukungiri, Kibaale, Hoima to Bundibugyo to Ntoroko); Kampala to Wakiso to Mukono peri‐urban cluster extending toward Masaka, Luweero, and Jinja; northern Lake Kyoga shoreline crescent (Apac, Kwania, Kole, and Kaberamaido)
SUDV	57	0.961	0.083	0.254 (25.4%)	Core belt (Bundibugyo to Ntoroko through Kagadi, Kibaale, and Hoima) with eastward extension into Kampala to Wakiso to Mukono; secondary arm along northern Lake Kyoga shoreline (Apac to Kwania to Oyam)
BDBV	7	0.999	0.524	0.003 (0.3%)	Tightly confined Albertine Rift escarpment focus (Ntoroko, Bundibugyo, Kabarole, Kyenjojo, Kikuube, Kanungu, and Rukungiri); small isolated nucleus over Kampala to Wakiso
EBOV	7	0.993	0.086	0.045 (about 4.5%)	Narrow western border ribbon within about 40 km of the Democratic Republic of the Congo border (Kisoro to Kanungu north through Rwenzori foothills including Kasese, Bundibugyo, Ntoroko into Nebbi and Arua); minimal inland extension

### 3.12. Alternative Partitioning Sensitivity

Sensitivity analysis using checkerboard2 partitioning produced a small increase in mean heldout AUC relative to spatial blocks (ΔAUC = +0.006) (Supporting Figure S3) and showed high agreement in four‐tier risk classification with most disagreement limited to adjacent tiers, supporting robustness of high‐tier suitability areas rankings to reasonable changes in the validation strategy (Supporting Figure S4).

## 4. Discussion

The MaxEnt ecological niche modeling, identified high relative suitability surfaces across Uganda with strong discriminatory accuracy. The model highlighted three primary high‐risk areas: the Albertine rift escarpment, the Kampala–Wakiso–Mukono peri‐urban corridor, and the Lake Kyoga northern shoreline. Human population density showed the strongest association with fitted cloglog suitability. Bushmeat activity and fruit‐bat roost density further defined the risk. Risk stratification classified about 6% of Uganda as tier 1, encompassing 42 districts and 29% of the population. Species‐specific spatial patterns were evident: SUDV suitability concentrated in peri‐urban zones such as Kampala–Wakiso, BDBV clustered tightly in the Albertine rift, and Zaire ebolavirus risk was confined to western border districts near the Democratic Republic of the Congo, aligning with cross‐border genomic imports. The model’s binary suitable‐area mask captured all historical outbreaks within an 8 km buffer, validating its spatial precision. These findings align with and expand upon a growing body of literature examining ecological and anthropogenic drivers of relative environmental suitability for reported Ebola spillover events, while offering evidence into Uganda’s spatially explicit risk landscape.

Suitability maps derived from reported spillover localities can reflect multiple processes beyond the underlying zoonotic hazard, including human outbreak amplification and the probability that an event is detected, confirmed, and reported. Presence‐only occurrence datasets are well known to be vulnerable to observer/sample‐selection bias when observations are more likely to be recorded in accessible or highly monitored locations, which can imprint surveillance and accessibility patterns onto inferred suitability [16]. Anthropogenic predictors such as population density are plausibly related to relative environmental suitability for reported Ebola spillover events through increased contact opportunities and outbreak propagation, but they can also serve as partial proxies for reporting intensity and health‐system access, complicating causal interpretation when surveillance effort varies spatially [16]. The dominant contribution of human population density should therefore be interpreted cautiously. Population density is not a direct ecological measure of reservoir presence or viral circulation. Instead, it likely integrates several linked but distinct processes: human exposure opportunity at the wildlife, land use, and settlement interface; the probability that an event is detected, diagnosed, and reported; and the potential for early outbreak amplification in more densely connected populations. Anthropogenic predictors, particularly population density, can therefore represent contact opportunity, early outbreak amplification, and surveillance access rather than ecological spillover hazard alone. We reduced this concern using three safeguards: health‐facility‐weighted background sampling [16], 1 km spatial thinning [15], and spatial block cross‐validation [24]. These steps reduce, but cannot eliminate, residual detection bias. We therefore interpret the mapped outputs as relative suitability surfaces for reported spillover localities and operational preparedness prioritization.

The spatial pattern of higher suitability is consistent with Uganda’s ecological gradients and land‐use mosaics that shape where human–wildlife contact is most likely and where habitat conditions can support persistence of potential reservoir hosts. Multiple lines of evidence implicate fruit bats in Ebolavirus ecology, including field evidence of asymptomatic infection and viral markers in several bat species in outbreak landscapes, although the specific mechanisms linking bat infection, shedding, and human index events can vary and remain incompletely resolved [2]. A mechanistic interpretation is plausible because climate seasonality and land‐surface conditions can modulate reservoir ecology and contact opportunities by influencing food availability, movement and aggregation, and stress‐related shedding dynamics in bats, as well as the timing and location of human activities such as farming, forest product collection, and hunting that mediate exposure [26]. Consistent with this, continental analyses have reported that Ebola spillover intensity is elevated during seasonal hydroclimatic transitions (wet–dry or dry–wet) in typically wet regions, supporting a role for seasonal dynamics in shaping spillover windows [27].

The concentration of high‐tier suitability areas near major settlement corridors is also ecologically plausible because land‐use change can increase forest–human edges and interface contact while simultaneously increasing the likelihood that spillover events are detected in dense populations. Large‐scale outbreak analyses have linked Ebola outbreak occurrence to recent loss of closed forest, suggesting that disturbance and fragmentation can contribute to relative environmental suitability for reported Ebola spillover events by altering human–wildlife interfaces [28]. These patterns support interpreting high‐tier suitability areas as reflecting the combined influence of ecological context (seasonal and habitat conditions relevant to reservoirs/hosts) and human exposure opportunity, reinforcing the value of integrating One Health surveillance with locally appropriate risk reduction and structured community engagement [29].

Human population density was the dominant predictor in the pooled model, but its interpretation is necessarily composite. Higher population density may increase human–wildlife contact opportunities at settlement, agricultural, market, and mobility interfaces [30, 31], while also increasing the chance that an event is detected, reported, and amplified after the index infection [32]. Similar population‐density signals have been reported in previous Ebola suitability analyses [33] and historical spillover reconstructions [27], but this convergence should not be read as evidence that population density is a direct ecological driver of viral circulation. Rather, it marks places where exposure opportunity, detection probability, and preparedness consequences overlap.

The hump‐shaped response of relative suitability surface to bat roost density (peaking at 0.08–0.12 km^−2^) underscores the role of chiropteran reservoirs in Ebola ecology. Fruit bats (family Pteropodidae) are established reservoirs for Ebola viruses [2], and the geographic overlap of their ranges with densely populated forest‐agriculture mosaics in western and central Uganda has been repeatedly implicated in spillover events, including the SUDV and BDBV outbreaks recorded since 2000 [3, 14]. The observed downturn in ecological suitability at very high bat densities (> 0.25 roosts km^−2^) is biologically plausible: dense, intact roost sites tend to lie deep inside undisturbed forest blocks that humans visit only sporadically, so the net human–bat contact rate and hence relative suitability can actually fall once bat abundance passes a certain threshold. Landscape‐scale modeling shows that zoonotic relative suitability for forest‐borne viruses is highest at intermediate levels of habitat loss or fragmentation and declines again inside large, continuous forest tracts, where edge density (the main contact arena) is minimal [34]. Uganda’s 2007 Marburg outbreak provides a concrete illustration of how rare, high‐contact incursions into otherwise secluded, high‐density colonies can nevertheless override that protective effect. Four gold miners working inside Kitaka Mine, a cavern harboring an estimated 40,000–1,00,000 Rousettus aegyptiacus bats, contracted Marburg virus after intensive subterranean exposure, confirming the colony as the infection source [35].

Precipitation seasonality (Bio15) and annual mean temperature (Bio1) made only modest contributions to our model yet this pattern echoes broader evidence that rain‐fall variability, rather than absolute temperature, is the dominant climatic trigger of relative environmental suitability for reported Ebola spillover localities. A multicountry landscape analysis found monthly rainfall the most sensitive climatic layer, whereas annual temperature had the weakest effect on suitability scores [36]. Event‐based modeling of 37 spillovers across Africa similarly showed peaks during transitions between wet and dry seasons, implicating precipitation seasonality as the immediate climatic “switch” for emergence [27]. Historic remote‐sensing work on the 1994–1996 outbreaks also linked abrupt shifts from drier to wetter conditions to index‐case timing [37].

Within Uganda, the stability of rainfall in mid‐elevation forests (≈ 800–1400 m) appears to maintain a year‐round fruit supply. Two decades of phenological monitoring at Ngogo, Kibale National Park, revealed relatively low month‐to‐month variability and sustained fruit production under buffered rainfall regimes [38], a pattern already noted in earlier multisite surveys of Kibale canopy trees [26]. Consistent fruit availability shapes bat ecology in ways that matter for virus dynamics. Seasonal pulses of Marburg virus shedding in Rousettus aegyptiacus colonies coincide with juvenile recruitment that is itself timed to resource peaks [39], while broader syntheses of bat‐virus systems show that such resource‐driven movements create windows of heightened shedding and spillover risk for filoviruses [26]. Collectively, these lines of evidence support our interpretation that moderate climatic covariates in the model capture a real, ecologically mediated link: stable, fruit‐sustaining rainfall regimes at Uganda’s mid‐elevations foster predictable bat foraging and breeding, which in turn modulate viral shedding and the timing of human spillover events.

The spatial segregation we observe between SUDV and BDBV is consistent with their distinct reservoir ecologies and the human activities that bring each virus into contact with people. SUDV outbreaks repeatedly ignite in Uganda’s densely settled commuter belt Luwero (2011), Mubende–Wakiso–Kampala (2022–23), and earlier peri‐urban clusters around Jinja where healthcare settings, funeral rites, and trading hubs provide multiple, person‐rich interfaces for onward spread [11, 40, 41]. BDBV, by contrast, has remained confined to the forested western flank of the Albertine rift. The 2007–2008 Bundibugyo outbreak began within remote villages bordering Semuliki National Park, and molecular tracing linked the virus to cave‐roosting Rousettus aegyptiacus fruit bats that dominate the local karst landscape [42, 43]. The same ecological interface dense bat colonies in little‐visited caves or abandoned mines matches other filovirus incursions in the rift system, underscoring how limited human access can keep BDBV’s niche spatially tight. A broader continental comparison reinforces this dichotomy: Zaire ebolavirus tends to emerge in sparsely populated, trans‐boundary forest blocks along the Uganda– Democratic Republic of the Congo frontier, whereas SUDV relative suitability trace Uganda’s east–west highway and labor‐migration corridor, mirroring human mobility more than strict biogeography [14, 44].

While virus‐specific suitability maps are useful for hypothesis generation and for guiding targeted ecological investigation, the interpretation depends strongly on sample size and on how “events” are defined and georeferenced. With very small numbers of occurrence localities, common performance statistics, especially AUC, can appear artificially high and may not reflect reliable geographic generalization, because evaluation becomes sensitive to extent, prevalence, and how background data are sampled [45]. In these small‐sample settings, controlling model complexity is essential: overly flexible feature sets can overfit idiosyncrasies of the limited occurrence data, reducing transferability and interpretability, while overly simplified models can miss important responses [46]. Practical small‐sample guidance therefore emphasizes parsimony and explicit tuning of feature classes and regularization, and when feasible, the use of small‐sample validation approaches alongside clear reporting of uncertainty [47].

Accordingly, virus‐specific maps for species represented by few localities should be interpreted as broad‐scale robustness checks and starting points for targeted field and surveillance hypotheses rather than fine‐scale prediction products.

The operational implication is that Tier 1 and Tier 2 areas should be treated as priority zones for strengthened preparedness, rather than as deterministic spillover‐risk zones. These areas are concentrated mainly along the Albertine Rift corridor, central peri‐urban corridor, and Lake Kyoga crescent, providing a practical basis for prioritizing enhanced event‐based surveillance, rapid alert verification, refresher training for health workers on EBOD case definitions, pre‐positioning of infection prevention and control supplies, rapid response team readiness, laboratory referral pathways, safe sample transport, and structured community engagement. These actions should be adapted using local epidemiological intelligence, district preparedness assessments, and ongoing One Health surveillance data.

The species‐specific suitability surfaces can further guide which interfaces should receive emphasis within these priority zones. For SUDV, where suitability extends into peri‐urban and settlement corridors, preparedness may focus on facility‐linked and event‐based detection, community reporting, and surveillance aligned with mobility and market networks. For BDBV, where suitability is more concentrated in forest‐edge landscapes, preparedness may place greater emphasis on One Health field investigations and community engagement around locally relevant forest, agriculture, and occupational contact interfaces. This interface‐specific approach is consistent with multisectoral, risk‐based prioritization at the human, animal, and environmental interface and with strengthening integrated surveillance and response capacities according to subnational risk profiles [48].

Several limitations should be considered. First, occurrence localities represent the most likely documented sites of initial human infection, not necessarily the exact sites of zoonotic transmission. Exposure histories may be incomplete or affected by recall and reporting bias, and residual positional uncertainty remains despite assigning geographic precision classes. The maps should therefore be interpreted more confidently at corridor and district‐prioritization scales than at exact point‐location scales.

Second, reported Ebola event localities may reflect detection and reporting geography as well as exposure geography. Health‐facility‐weighted background sampling was used to reduce this bias, but it cannot fully capture variation in surveillance intensity, diagnostic capacity, specimen referral, community alert behavior, or outbreak‐specific reporting conditions.

Third, several predictors were proxies rather than direct measurements of biological processes. Wildlife‐related layers may be affected by uneven sampling and incomplete coverage, while human population density may reflect exposure opportunity, detection/reporting probability, and early outbreak amplification rather than a direct ecological driver of zoonotic transmission.

Fourth, the MaxEnt suitability surfaces represent relative modeled suitability and should not be interpreted as calibrated probabilities of Ebola spillover or direct measures of absolute zoonotic hazard. The maps are intended as operational prioritization tools for surveillance and preparedness.

Fifth, the health‐facility bias surface is an imperfect proxy for detection and reporting probability. Facility density does not capture all determinants of surveillance sensitivity, including facility capacity, diagnostic readiness, specimen transport, community alert behavior, cross‐border mobility, or temporal changes in surveillance intensity. It is therefore interpreted as a reproducible approximation of relative reporting opportunity, and residual detection bias may remain.

Finally, the species‐specific models, especially BDBV and Zaire ebolavirus, require cautious interpretation because they were fitted using very small occurrence subsets and may have inflated training AUC values. We also did not conduct a population‐density exclusion sensitivity analysis or variance partitioning; therefore, the dominant population‐density signal should be interpreted as a composite indicator of exposure, detection, and amplification processes rather than a direct ecological driver.

## 5. Conclusion

This study generated 1 km of pooled and virus‐specific relative suitability surfaces for reported EBOD spillover localities in Uganda and translated the pooled output into a four‐tier framework for preparedness planning. The highest‐priority areas were concentrated in the Albertine Rift escarpment belt, complemented by a peri‐urban cluster around Kampala–Wakiso–Mukono extending along major settlement corridors and the northern Lake Kyoga crescent, where high modeled suitability overlaps with substantial resident populations. These findings provide a practical prioritization framework for EBOD preparedness in Uganda. Tier 1 and Tier 2 areas, particularly along the Albertine Rift corridor, the central peri‐urban corridor, and the Lake Kyoga crescent, represent priority zones where enhanced event‐based surveillance, early detection and reporting systems, pre‐positioned infection prevention and control supplies, trained rapid response teams, laboratory referral pathways, and community engagement may be most impactful. Because the maps represent relative suitability for reported Ebola spillover localities rather than direct measurements of absolute spillover probability, they should be used alongside local epidemiological intelligence, district preparedness assessments, and One Health surveillance systems.

## Author Contributions

Conceptualization: G.P. and P.O‐O. Data curation: N.M. and G.P. Methodology and formal analysis: G.P., N.M., and S.O. Visualization: G.P. and N.M. Validation: S.O. and P.O‐O. Writing–original draft: G.P. Writing–review and editing: S.O., N.M., and P.O.O.

## Funding

The authors received no financial support for the research, authorship, and/or publication of this article.

## Disclosure

All authors read and approved the final manuscript. An earlier version of the manuscript was posted online as a preprint in Research Square [49].

## Ethics Statement

All reported Ebola spillover localities’ coordinates were abstracted from publicly available outbreak line lists and peer‐reviewed literature; no human subjects or identifiable patient data were used. Consequently, the study was granted a waiver of informed consent by the Mbale Regional Referral Hospital, Research and Ethics Committee (MRRH‐2025‐607).

## Consent

Please see the Ethics Statement.

## Conflicts of Interest

The authors declare no conflicts of interest.

## Supporting Information

Additional supporting information can be found online in the Supporting Information section.

## Supporting information


**Supporting Information 1** Supporting File 1 is the ISLE‐ReSt checklist completed for this study, indicating where each reporting item is addressed in the manuscript.

**Supporting Information 2** Supporting File 2 contains additional Supporting Methods, Supporting Tables, and Supporting Figures, including detailed occurrence database construction and georeferencing procedures, health‐facility bias surface construction, predictor inventory and 1 km processing workflow, bat‐density proxy construction, multicollinearity screening workflow, and the full tuning and fold‐wise evaluation outputs (including > Table S1–S5 and > Figure S2–S4).

## Data Availability

The complete workflow, georeferenced reported Ebola spillover localities (with spatial unit (villages/parishes/subcounties) centroids jittered by ±2 km to protect privacy), final 1‐km GeoTIFF risk rasters, and R markdown notebooks are archived on Zenodo (https://doi.org/10.5281/zenodo.15600735) and mirrored on GitHub (https://github.com/gpaasi/ebola-MaxEnt-ecological-niche-modeling-uganda).
