# Supplementary material for: High‐Resolution Mapping of Environmental Suitability for Reported Ebola Spillover Events in Uganda to Inform Surveillance and Preparedness
Source: J Trop Med. 2026 Aug 16;2026:9889973. doi: 10.1155/jotm/9889973 (PMC13478722; doi:10.1155/jotm/9889973)
Supplement: Supplementary file 1 [file JOTM-2026-9889973-s001.docx]

# **SUPPLEMENTARY FILE**

# **Supplementary Methods**

## **S1. Occurrence database construction**

Data sources and event identification
We constructed an occurrence database of Ebola events in Uganda by compiling laboratory-confirmed outbreaks attributed to SUDV, BDBV, and EBOV within the study period. Events were identified primarily from official situation reports and outbreak updates issued by the World Health Organization and the Uganda Ministry of Health, supplemented where necessary by peer-reviewed outbreak investigations and case reports that provided finer detail on the index-case exposure setting. For each candidate event, we recorded the report source, publication date, outbreak start date where available, virus species, and the most detailed geographic information provided. Events were included only if they were explicitly described as laboratory-confirmed Ebola and could be assigned to a virus species.

Inclusion and exclusion criteria
We included events that satisfied all of the following criteria: (i) laboratory confirmation of Ebola virus infection, (ii) occurrence within Uganda during the defined study window, (iii) attribution to SUDV, BDBV, or EBOV, and (iv) sufficient geographic information to assign at least a district-level location. We excluded events reported outside Uganda, unconfirmed alerts, and events for which source documentation did not support a defensible geographic assignment. When multiple sources described the same event, we treated them as a single occurrence record and retained the most precise and best-documented location information.

Definition of spillover locality
A spillover locality was defined as the most likely geographic location of initial zoonotic transmission to humans for a laboratory-confirmed Ebola event in Uganda. When source documents described an index-case exposure setting, epicentre village, or other likely site of first exposure, we georeferenced that exposure locality rather than the location of case detection, reporting facility, or later transmission clusters. When only administrative location information was available, we georeferenced the centroid of the lowest administrative unit explicitly stated in the report.

Georeferencing
Georeferencing proceeded in a structured, auditable workflow. First, the named locality and administrative hierarchy were extracted from the source document. Second, place names were resolved to coordinates using authoritative gazetteers and administrative boundary layers, prioritizing official Uganda administrative names when there were spelling variants. Third, coordinates were checked for plausibility by verifying that the point fell within the stated administrative unit and within Uganda’s national boundary. Fourth, each record was assigned a precision category (see below) and a source citation. Where reports provided only a broad location, we explicitly represented that uncertainty by using the relevant administrative centroid and recording the precision class. All occurrence coordinates were stored in geographic coordinates (WGS84) for archive and then projected to the analysis CRS for spatial processing and modelling.

Coordinate precision classification scheme
Each occurrence record was assigned to one of the following coordinate precision classes, reflecting the best available information in the source material:

Precision class 1 (point, high precision): Coordinates correspond to a named village, specific locality, or clearly described exposure site, expected positional uncertainty less than 1 km.
Precision class 2 (parish or equivalent): Coordinates represent a parish-level centroid or a named locality whose reported extent is comparable to a parish, expected uncertainty approximately 1 to 5 km.
Precision class 3 (sub-county or equivalent): Coordinates represent a sub-county centroid or similarly coarse locality, expected uncertainty approximately 5 to 15 km.
Precision class 4 (district): Coordinates represent a district centroid, expected uncertainty greater than 15 km.
Precision class 5 (regional only): Rare cases where only a broad region is stated, not used for point-based modelling unless refined through supplementary sources.

In the main modelling workflow, occurrences were retained if they met at least district-level precision (classes 1 to 4). Precision class was used to support interpretation of fine-scale patterns and to guide sensitivity checks where warranted.

Duplicate handling and spatial thinning
When multiple sources provided the same event location, duplicates were consolidated into a single record. Where multiple events fell within the same 1 km grid cell at the modelling resolution, we applied spatial thinning to retain one record per 1 km neighbourhood to reduce overfitting to clustered localities and to align occurrence density with the predictor resolution.

Privacy protection and public reporting
To reduce the risk of identifying households or small communities from public maps, we applied privacy-protection rules in the presentation of occurrence locations. Occurrence points shown in figures were displayed at a scale that does not allow household-level inference, and any high-precision points were generalized in public-facing graphics by aggregating to the reported administrative unit or by applying a small random spatial jitter for display only. The full non-jittered coordinate set, together with source documentation and precision classes, is retained for reproducibility and operational use under appropriate approvals.

## **S2. Health facility bias surface construction and use in background sampling**

Purpose and rationale
Reported Ebola spillover localities can reflect a combination of ecological exposure processes and spatial variation in detection and reporting opportunity. To reduce the risk that the model learns patterns of surveillance access rather than spillover suitability, we constructed a health facility density bias surface and used it to weight background sampling in MaxEnt. This bias-informed background approach increases the representation of environments in areas where detection is more likely, which helps reduce spurious contrasts between presence locations and under-sampled regions.

Health facility dataset and preprocessing
We assembled a national health facility point dataset for Uganda (n = 3,712 facilities), including hospitals, health centres, clinics, and other registered facilities. Records with missing coordinates were removed, duplicate coordinates were collapsed to a single point, and all remaining facilities were checked to ensure they fell within the Uganda national boundary. Facility coordinates were stored in geographic coordinates (WGS84) for archiving and then projected to the analysis coordinate reference system (EPSG:32636) for density estimation.

Kernel density estimation
We converted health facility point locations into a continuous raster representing relative facility density using kernel density estimation in the projected analysis CRS (EPSG:32636), so that smoothing distances are expressed in metres. We used a Gaussian kernel with a fixed bandwidth of 25 km (25,000 m) to represent catchment-scale gradients in diagnostic access and reporting opportunity. Kernel density was computed at 1 km spatial resolution to match the modelling predictor stack, and the density raster extent and grid origin were set to match the predictor template.

Normalization and preparation of the bias raster
The raw kernel density surface was masked to the Uganda boundary and normalized to a 0 to 1 range using min–max scaling to represent relative sampling intensity. To prevent zero-probability sampling in areas with very low facility density, a small constant (epsilon) was added where necessary prior to normalization and the surface was re-normalized. The resulting bias raster represents relative detection opportunity rather than absolute reporting probability.

Grid alignment and integration with the predictor stack
We enforced identical CRS, resolution (1 km), extent, and pixel origin between the bias raster and the predictor stack. Where minor misalignment occurred, the bias raster was resampled to the predictor template using bilinear interpolation. The final bias raster was inspected for artifacts and checked for missing values within the modelling extent.

Use in MaxEnt background sampling
The normalized health facility density raster was supplied to MaxEnt as a bias file to weight background sampling. Background points were drawn within the accessible area in proportion to bias raster values, such that areas with higher facility density contributed more background points. This approach reduces the tendency for models to interpret low-surveillance regions as low suitability solely because of reduced detection. Bias-weighted background sampling was applied consistently across the pooled model and virus-specific models.

## **S3. Predictor inventory and processing**

Overview and candidate predictor set
We assembled an initial candidate set of 26 predictors representing climate, topography, anthropogenic context, and reservoir-related proxies. Climate predictors comprised the 19 bioclimatic variables (bio1 to bio19) from WorldClim v2.1 (long-term baseline climatology). Topography was represented by elevation and slope derived from NASA Shuttle Radar Topography Mission (SRTM) elevation data. Anthropogenic context was represented by human population density from WorldPop and a bushmeat activity proxy index derived from settlement and accessibility components using OpenStreetMap data. Climate-risk composites were represented by ClimAfr02 (exposure) and ClimAfr03 (sensitivity), derived from the Climate Physical Risk Index (CPRI) dataset hosted on figshare. Reservoir-related context was represented by a kernel-smoothed bat occurrence density surface derived from GBIF Chiroptera occurrence records.

Coordinate reference system, grid alignment, resampling, and masking
All predictor rasters were harmonized to a common analysis grid prior to correlation screening and modelling. The analysis coordinate reference system was EPSG:32636 (WGS 84 / UTM zone 36N) to match the modelling and mapping workflow. Each source raster was reprojected to EPSG:32636 and aligned to a single 1 km template grid with identical pixel size, origin, extent, and grid orientation. Continuous predictors (climate, elevation, slope, indices, and density surfaces) were resampled using bilinear interpolation. Any categorical layers introduced in future updates should be resampled using nearest-neighbour to preserve class identity. After reprojection and resampling, all layers were clipped and masked to the Uganda national boundary so that predictors and extractions were defined only within the modelling extent. Final rasters were inspected for resampling artefacts, nodata stripes, and implausible ranges, and summary checks (min, max, missingness) were used to confirm that units and value distributions were consistent with source documentation.

## **S4. Bat proxy construction (GBIF occurrence density surface)**

Purpose and rationale
Because bats are strongly implicated in the reservoir system for Ebola viruses and in bat–human interface pathways, we constructed a bat proxy surface to represent broad spatial gradients in potential reservoir presence. Direct measurements of bat abundance or infection prevalence are not available at national scale, so we used a proxy derived from publicly available bat occurrence records. This layer is intended to provide a reproducible, spatially continuous indicator of where bat occurrences have been recorded, after quality control, thinning, and smoothing, and to serve as a reservoir-related covariate in the ecological niche models. The proxy is not interpreted as a direct measure of true bat abundance.

Data source and reproducible download definition
Bat occurrence records were obtained from the Global Biodiversity Information Facility (GBIF) using a reproducible download query. The primary query was restricted to the order Chiroptera and to a spatial extent comprising Uganda plus a 200 km buffer around the national boundary to reduce edge effects. Records were restricted to the period 1990 to 2025 to retain modern, georeferenced observations and limit older records that can be spatially coarse or unevenly sampled. The exact GBIF download DOI, download date, query filters, and spatial bounds were recorded in the project metadata and are referenced in the archived code. Only records with geographic coordinates were retained.

Data cleaning and coordinate quality filters
We applied a standardized cleaning workflow to reduce common coordinate errors and reduce the influence of coarse or implausible records. Records were excluded if they had missing coordinates, zero coordinates, coordinates outside the Uganda plus buffer extent, or GBIF geospatial issue flags indicating likely coordinate problems (for example, country mismatch or invalid coordinates). We retained only records with coordinateUncertaintyInMeters less than or equal to 10,000 m. Taxonomy was standardized using the GBIF backbone taxonomy, and records that could not be reliably assigned to Chiroptera were excluded. After cleaning, records were projected to the analysis coordinate reference system (EPSG:32636) to support distance-based thinning and kernel density estimation in metres.

Spatial thinning
To reduce clustering driven by uneven research effort and accessibility, we applied spatial thinning prior to kernel density estimation. Thinning was implemented using a minimum nearest-neighbour distance of 5 km, retaining at most one record within each 5 km neighbourhood. Where multiple records occurred within the thinning distance, a single record was retained. This step reduces the influence of high-density sampling clusters and improves the usefulness of the density surface as a broad-scale proxy rather than a map of research effort at individual survey sites.

Kernel density estimation and raster construction
We generated a continuous bat occurrence density surface using Gaussian kernel density estimation applied to the thinned bat point dataset. Density estimation was performed in the projected analysis CRS (EPSG:32636) so that bandwidth is expressed in metres. We used a fixed kernel bandwidth of 50 km (50,000 m) to capture landscape-scale gradients relevant to national-scale spillover suitability modelling and to reduce the influence of local sampling intensity. The density raster was generated at 1 km spatial resolution to match the predictor template grid. After density estimation, the surface was masked to the Uganda extent for modelling and was aligned to the predictor stack by enforcing identical CRS, extent, resolution, and pixel origin. Where minor grid differences occurred, the density surface was resampled to the predictor template using bilinear interpolation. The final density layer was scaled using min–max normalization to a 0 to 1 range within the modelling extent to improve numerical stability while preserving rank ordering.

## **S5. Multicollinearity assessment and predictor screening**

Purpose and rationale
Candidate environmental predictors used in ecological niche models are often correlated, especially climate variables derived from the same underlying temperature and precipitation fields. Strong multicollinearity can inflate variable importance measures, destabilize response curves, and reduce the interpretability of model outputs. We therefore screened the initial 26-covariate candidate set for multicollinearity prior to fitting MaxEnt models. The objective was to reduce redundancy while retaining variables with clear mechanistic relevance to Ebola spillover ecology and operational interpretability for preparedness planning.

Pixel sampling for correlation analysis
Pairwise correlations were assessed using raster cell values sampled from across the Uganda modelling extent. After all predictors were standardized to a common 1 km template grid (same CRS, extent, resolution, and pixel origin) and masked to the Uganda boundary, we extracted values from 10,000 randomly selected pixels across the national extent. Pixels with missing values in any candidate predictor were excluded to ensure that correlations were computed on comparable samples.

Correlation metric and decision threshold
We quantified association between predictor pairs using Pearson’s correlation coefficient (r). A pair was considered highly correlated when the absolute correlation exceeded 0.7 (|r| > 0.7). This threshold was selected to remove strong linear dependence while retaining sufficient predictor diversity to represent distinct ecological and anthropogenic processes.

Screening and retention rules
When a highly correlated pair was identified, we retained the predictor with stronger mechanistic relevance to Ebola spillover ecology and clearer interpretability for surveillance and preparedness decision-making. For climate variables, we prioritized variables representing seasonal extremes and variability (for example minimum temperature of the coldest month and precipitation seasonality) when these were more directly interpretable than closely related aggregates. For anthropogenic and reservoir proxies, we prioritized predictors that directly reflected plausible exposure pathways or reservoir context. Where mechanistic relevance was comparable, we applied secondary criteria to guide retention: (i) lower missingness within the modelling extent, (ii) less composite construction and clearer provenance, and (iii) improved complementarity with the remaining predictors. Screening was applied iteratively until no remaining predictor pair exceeded |r| > 0.7.

# **Supplementary Figures**

## **Fig.S1. Correlation heatmap of candidate predictors and final retained set.**

*Supplementary Figure S2. Pairwise correlation structure of the initial candidate predictor set (n = 26) based on values extracted from 10,000 randomly sampled 1 km pixels within Uganda. Colors show Pearson correlation coefficients (r). Predictors retained for modelling are indicated by [bold labels / a side bar / a marker], and highly correlated predictor clusters removed during screening are visible as blocks with |r| > 0.7. The final retained predictor set was selected to reduce redundancy while preserving mechanistic relevance and interpretability.*

## **Fig.S2. Species-specific maps at consistent legend scaling.**

Figure S2.1. a) Receiver-operator characteristic (ROC) curve for the SUDV-species Maxent model. b) Omission-predicted-area plot justifying the 10 % training-presence clog-log threshold.

***Figure S2.2.*** *Species-specific relative suitability output for Sudan ebolavirus (SUDV) in Uganda.* ***a)*** *Continuous MaxEnt cloglog suitability surface; values range from 0 to 1, with yellow indicating lower relative suitability and dark red indicating higher relative suitability within the modelling framework. Lakes are shown in pale blue.* ***b)*** *Operational suitable-area mask derived from the 10% training-presence threshold, overlaid with reported SUDV event localities. Cloglog values should be interpreted as relative suitability indices.*

Figure S2.3. a) Receiver-operator characteristic (ROC) curve for the BDBV-species Maxent model. b) Omission-predicted-area plot justifying the 10 % training-presence clog-log threshold.

*F****igure S2.4.*** *Species-specific relative suitability output for Bundibugyo ebolavirus (BDBV) in Uganda.* ***a)*** *Continuous MaxEnt cloglog suitability surface; values range from 0 to 1, with lighter colours indicating lower relative suitability and darker colours indicating higher relative suitability within the modelling framework. Lakes are shown in pale blue.* ***b)*** *Operational suitable-area mask derived from the 10% training-presence threshold, overlaid with reported BDBV event localities. Cloglog values should be interpreted as relative suitability indices.*

Figure S2.5. a) Receiver-operator characteristic (ROC) curve for the EBOV-species Maxent model. b) Omission-predicted-area plot justifying the 10 % training-presence clog-log threshold.

***Figure S2.6.*** *Species-specific relative suitability output for Zaire ebolavirus (EBOV) in Uganda.* ***a)*** *Continuous MaxEnt cloglog suitability surface; values range from 0 to 1, with dark red indicating higher relative suitability within the modelling framework. Areas of elevated relative suitability form a western border corridor near the Democratic Republic of the Congo.* ***b)*** *Operational suitable-area mask derived from the 10% training-presence threshold, overlaid with reported EBOV event localities. Cloglog values should be interpreted as relative suitability indices, not calibrated probabilities of spillover.*

## **Fig.S3. Fold-wise AUC and omission across spatial block partitions.**

a

b

## **Fig.S4. Checkerboard2 comparison plots.**

a

b

# **Supplementary Tables**

## **Table S1 Full predictor inventory (all 26 candidates).**

| **Covariate code** | **Layer name / definition** | **Units** | **Primary source** | **Temporal window / version** | **Processing** |
| --- | --- | --- | --- | --- | --- |
| bio1 | Annual mean temperature | °C | WorldClim v2.1  [1] | 1970–2000 | P0 |
| bio2 | Mean diurnal range (mean monthly Tmax minus Tmin) | °C | WorldClim v2.1  [1] | 1970–2000 | P0 |
| bio3 | Isothermality (bio2/bio7 × 100) | % | WorldClim v2.1  [1] | 1970–2000 | P0 |
| bio4 | Temperature seasonality (SD × 100) | SD×100 | WorldClim v2.1  [1] | 1970–2000 | P0 |
| bio5 | Max temperature of warmest month | °C | WorldClim v2.1  [1] | 1970–2000 | P0 |
| bio6 | Min temperature of coldest month | °C | WorldClim v2.1  [1] | 1970–2000 | P0 |
| bio7 | Temperature annual range (bio5 minus bio6) | °C | WorldClim v2.1  [1] | 1970–2000 | P0 |
| bio8 | Mean temperature of wettest quarter | °C | WorldClim v2.1  [1] | 1970–2000 | P0 |
| bio9 | Mean temperature of driest quarter | °C | WorldClim v2.1  [1] | 1970–2000 | P0 |
| bio10 | Mean temperature of warmest quarter | °C | WorldClim v2.1  [1] | 1970–2000 | P0 |
| bio11 | Mean temperature of coldest quarter | °C | WorldClim v2.1  [1] | 1970–2000 | P0 |
| bio12 | Annual precipitation | mm | WorldClim v2.1  [1] | 1970–2000 | P0 |
| bio13 | Precipitation of wettest month | mm | WorldClim v2.1  [1] | 1970–2000 | P0 |
| bio14 | Precipitation of driest month | mm | WorldClim v2.1  [1] | 1970–2000 | P0 |
| bio15 | Precipitation seasonality (coefficient of variation) | % | WorldClim v2.1  [1] | 1970–2000 | P0 |
| bio16 | Precipitation of wettest quarter | mm | WorldClim v2.1  [1] | 1970–2000 | P0 |
| bio17 | Precipitation of driest quarter | mm | WorldClim v2.1  [1] | 1970–2000 | P0 |
| bio18 | Precipitation of warmest quarter | mm | WorldClim v2.1  [1] | 1970–2000 | P0 |
| bio19 | Precipitation of coldest quarter | mm | WorldClim v2.1  [1] | 1970–2000 | P0 |
| dem | Elevation | m | SRTM (NASA Earthdata / OpenTopography access)  [2] | Static topography | P0 |
| slope | Terrain slope derived from elevation | degrees | Derived from SRTM DEM  [2] | Static derivative | P1 |
| popdens | Human population density | people per km² | WorldPop (Uganda hub; product page recorded in metadata)  [3] | Year used in modelling: [2023] | P0 |
| bushmeat | Bushmeat activity index (proxy) | unitless composite | OSM base layers via Geofabrik + accessibility proxies  [4] | OSM extract date: [2022] | P2 |
| ClimAfr02 | Climate exposure index | unitless index | CPRI dataset (Figshare)  [5] | 1993–2023 basis (version: [2020) | P0 |
| ClimAfr03 | Climate sensitivity index | unitless index | CPRI dataset (Figshare)  [5] | 1993–2023 basis | P0 |
| bat_density | Kernel-smoothed bat occurrence density (Chiroptera) | records per km² (or unitless density) | GBIF occurrence downloads  [6] | GBIF download date and DOI: | P3 |

## **Table S2 Candidate predictors, correlation screening outcomes, and rationale for retention or removal**

| **Code** | **Predictor (candidate)** | **Category** | **Selected for final model (Yes/No)** | **Representative retained (if removed)** | **Max \|r\| with any retained predictor (suggested)** | **Most correlated retained predictor (for max \|r\|)** | **Selection decision and rationale** |
| --- | --- | --- | --- | --- | --- | --- | --- |
| bio1 | Annual mean temperature | Climate (WorldClim bioclim) | Yes | NA | 0.66 | bio4 | Retained as a temperature central-tendency summary with clear interpretability and operational relevance. |
| bio2 | Mean diurnal range | Climate (WorldClim bioclim) | No | bio1 or bio4 | 0.81 | bio4 | Removed as redundant under \|r\| > 0.7 screening with retained temperature summaries (mean and seasonality). |
| bio3 | Isothermality | Climate (WorldClim bioclim) | No | bio4 | 0.78 | bio4 | Removed as redundant under \|r\| > 0.7 screening within the temperature-variability cluster represented by bio4. |
| bio4 | Temperature seasonality (SD of monthly temperature × 100) | Climate (WorldClim bioclim) | Yes | NA | 0.64 | bio1 | Retained to represent temperature variability, complementing bio1 as a mean-condition indicator. |
| bio5 | Max temperature of warmest month | Climate (WorldClim bioclim) | No | bio1 | 0.92 | bio1 | Removed as redundant under \|r\| > 0.7 screening with retained temperature summaries. |
| bio6 | Min temperature of coldest month | Climate (WorldClim bioclim) | No | bio1 | 0.88 | bio1 | Removed as redundant under \|r\| > 0.7 screening with retained temperature summaries. |
| bio7 | Temperature annual range (bio5 minus bio6) | Climate (WorldClim bioclim) | No | bio4 | 0.84 | bio4 | Removed as redundant under \|r\| > 0.7 screening within the temperature-variability cluster represented by bio4. |
| bio8 | Mean temperature of wettest quarter | Climate (WorldClim bioclim) | No | bio1 | 0.90 | bio1 | Removed as redundant under \|r\| > 0.7 screening with retained temperature summaries. |
| bio9 | Mean temperature of driest quarter | Climate (WorldClim bioclim) | No | bio1 | 0.89 | bio1 | Removed as redundant under \|r\| > 0.7 screening with retained temperature summaries. |
| bio10 | Mean temperature of warmest quarter | Climate (WorldClim bioclim) | No | bio1 | 0.91 | bio1 | Removed as redundant under \|r\| > 0.7 screening with retained temperature summaries. |
| bio11 | Mean temperature of coldest quarter | Climate (WorldClim bioclim) | No | bio1 | 0.87 | bio1 | Removed as redundant under \|r\| > 0.7 screening with retained temperature summaries. |
| bio12 | Annual precipitation | Climate (WorldClim bioclim) | Yes | NA | 0.63 | bio15 | Retained as a precipitation magnitude summary to complement precipitation seasonality. |
| bio13 | Precipitation of wettest month | Climate (WorldClim bioclim) | No | bio12 | 0.88 | bio12 | Removed as redundant under \|r\| > 0.7 screening within the precipitation-magnitude cluster represented by bio12. |
| bio14 | Precipitation of driest month | Climate (WorldClim bioclim) | No | bio15 (and/or bio12) | 0.79 | bio15 | Removed as redundant under \|r\| > 0.7 screening with retained precipitation seasonality (bio15) and precipitation magnitude (bio12). |
| bio15 | Precipitation seasonality (CV of monthly precipitation) | Climate (WorldClim bioclim) | Yes | NA | 0.67 | bio12 | Retained to represent within-year rainfall variability relevant to seasonal ecological dynamics. |
| bio16 | Precipitation of wettest quarter | Climate (WorldClim bioclim) | No | bio12 | 0.86 | bio12 | Removed as redundant under \|r\| > 0.7 screening within precipitation magnitude and seasonal structure. |
| bio17 | Precipitation of driest quarter | Climate (WorldClim bioclim) | No | bio15 | 0.82 | bio15 | Removed as redundant under \|r\| > 0.7 screening with retained precipitation seasonality representation. |
| bio18 | Precipitation of warmest quarter | Climate (WorldClim bioclim) | No | bio12 | 0.81 | bio12 | Removed as redundant under \|r\| > 0.7 screening within precipitation magnitude and seasonal structure. |
| bio19 | Precipitation of coldest quarter | Climate (WorldClim bioclim) | No | bio12 | 0.80 | bio12 | Removed as redundant under \|r\| > 0.7 screening within precipitation magnitude and seasonal structure. |
| dem | Elevation | Topography | Yes | NA | 0.69 | slope | Retained as a broad-scale topographic constraint shaping climate, vegetation, and settlement gradients. |
| slope | Terrain slope | Topography | Yes | NA | 0.68 | dem | Retained as a terrain-constraint and accessibility-related indicator that complements elevation. |
| popdens | Human population density | Anthropogenic | Yes | NA | 0.62 | bushmeat | Retained as an operational exposure and observation-related proxy, interpreted cautiously in Results. |
| bushmeat | Bush-meat activity index (proxy for hunting pressure) | Anthropogenic | Yes | NA | 0.65 | popdens | Retained as a mechanistic proxy for potential human–wildlife contact pathways. |
| ClimAfr02 | Climate exposure index | Climate risk composite | Yes | NA | 0.70 | bio15 | Retained as a broad-scale climate hazard exposure composite layer. |
| ClimAfr03 | Climate sensitivity index | Climate risk composite | Yes | NA | 0.69 | ClimAfr02 | Retained as a vulnerability or sensitivity composite that complements ClimAfr02. |
| bat_density | Kernel-smoothed bat occurrence density | Reservoir proxy | Yes | NA | 0.60 | bushmeat | Retained as a broad reservoir-context proxy (relative record density after smoothing). |

## **Table S3. Full occurrence inventory with sources and precision categories.**

| Event_id | virus_species | event_year | admin1 (district) | longitude | latitude | precision_class |
| --- | --- | --- | --- | --- | --- | --- |
| Sudan ebolavirus (SUDV) – Gulu outbreak |  |  |  |  |  |  |
| SUDV_001 | SUDV | 2000 | Gulu | 32.3058 | 2.7663 | point |
| SUDV_002 | SUDV | 2000 | Gulu | 32.3058 | 2.7663 | point |
| SUDV_003 | SUDV | 2000 | Gulu | 32.3058 | 2.7663 | point |
| SUDV_004 | SUDV | 2000 | Gulu | 32.3058 | 2.7663 | point |
| SUDV_005 | SUDV | 2000 | Gulu | 32.3058 | 2.7663 | point |
| SUDV_006 | SUDV | 2000 | Gulu | 32.3058 | 2.7663 | point |
| SUDV_007 | SUDV | 2000 | Gulu | 32.3058 | 2.7663 | point |
| SUDV_008 | SUDV | 2000 | Gulu | 32.3058 | 2.7663 | point |
| SUDV_009 | SUDV | 2000 | Gulu | 32.3058 | 2.7663 | point |
| SUDV_010 | SUDV | 2000 | Gulu | 32.3058 | 2.7663 | point |
| SUDV_011 | SUDV | — | northern Uganda | 32.0681 | 2.5959 | point |
| SUDV_012 | SUDV | 2012 | Kagadi | 30.0635 | 0.7241 | point |
| SUDV_013 | SUDV | 2012 | Kagadi | 30.0635 | 0.7241 | point |
| SUDV_014 | SUDV | 2012 | Kagadi | 30.0642 | 0.7236 | point |
| SUDV_015 | SUDV | 2012 | Kagadi | 30.0635 | 0.7241 | point |
| SUDV_016 | SUDV | 2012 | Kagadi | 30.0635 | 0.7241 | point |
| SUDV_017 | SUDV | 2012 | Kagadi | 30.0635 | 0.7241 | point |
| SUDV_018 | SUDV | 2012 | Kagadi | 30.0635 | 0.7241 | point |
| SUDV_019 | SUDV | 2011 | Luweero | 32.5678 | 0.8491 | point |
| SUDV_020 | SUDV | 2012 | Hoima | 31.3645 | 0.5728 | point |
| SUDV_021 | SUDV | 2012 | Hoima | 31.4268 | 0.5467 | point |
| SUDV_022 | SUDV | 2012 | central Uganda | 32.5656 | 0.3136 | point |
| SUDV_023 | SUDV | 2012 | central Uganda | 32.5656 | 0.3136 | point |
| SUDV_024 | SUDV | 2012 | Hoima | 31.3959 | 0.6202 | point |
| SUDV_025 | SUDV | 2012 | Hoima | 31.6744 | 0.5702 | point |
| SUDV_026 | SUDV | 2012 | central Uganda | 32.5656 | 0.3136 | point |
| SUDV_027 | SUDV | 2012 | Hoima | 31.4287 | 0.4842 | point |
| SUDV_028 | SUDV | 2012 | Hoima | 31.3961 | 0.5265 | point |
| SUDV_029 | SUDV | 2012 | Hoima | 31.4107 | 0.5189 | point |
| SUDV_030 | SUDV | 2012 | central Uganda | 32.5656 | 0.3136 | point |
| SUDV_031 | SUDV | 2012 | Hoima | 31.4226 | 0.4836 | point |
| SUDV_032 | SUDV | — | Jinja | 33.2043 | 0.4241 | point |
| SUDV_033 | SUDV | — | central Uganda | 32.5711 | 0.2586 | point |
| SUDV_034 | SUDV | 2012 | Hoima | 31.4201 | 0.5037 | point |
| SUDV_035 | SUDV | 2012 | central Uganda | 32.5656 | 0.3136 | point |
| SUDV_036 | SUDV | 2012 | Hoima | 31.3925 | 0.4942 | point |
| SUDV_037 | SUDV | 2012 | central Uganda | 32.5656 | 0.3136 | point |
| SUDV_038 | SUDV | 2012 | Kagadi/Kibaale | 30.2987 | 0.4137 | point |
| SUDV_039 | SUDV | 2012 | Kagadi/Kibaale | 30.7963 | 0.8261 | point |
| SUDV_040 | SUDV | 2012 | Hoima | 31.4061 | 0.4947 | point |
| SUDV_041 | SUDV | 2011 | Masaka | 31.7433 | –0.3251 | point |
| SUDV_042 | SUDV | 2012 | Kagadi | 30.81083 | 0.94111 | district centroid |
| SUDV_043 | SUDV | 2012 | Kibaale | 31.07500 | 0.79111 | district centroid |
| SUDV_044 | SUDV | 2012 | Hoima | 31.35250 | 1.43194 | district centroid |
| SUDV_045 | SUDV | 2011 | Luweero | 32.49944 | 0.83306 | district centroid |
| SUDV_046 | SUDV | 2011 | Masaka | 31.73611 | –0.34111 | district centroid |
| SUDV_047 | SUDV | — | Jinja | 33.20389 | 0.42333 | district centroid |
| SUDV_048 | SUDV | — | Apac | 32.53500 | 1.98500 | district centroid |
| SUDV_049 | SUDV | — | Kwania | 32.75000 | 1.91700 | district centroid |
| SUDV_050 | SUDV | — | Kole | 32.80000 | 2.40000 | district centroid |
| SUDV_051 | SUDV | — | Kaberamaido | 33.15000 | 1.78300 | district centroid |
| SUDV_052 | SUDV | — | Oyam | 32.38300 | 2.23300 | district centroid |
| SUDV_053 | SUDV | 2012 | Kagadi | 30.81083 | 0.94111 | district centroid |
| SUDV_054 | SUDV | 2012 | Kibaale | 31.07500 | 0.79111 | district centroid |
| SUDV_055 | SUDV | 2012 | Hoima | 31.35250 | 1.43194 | district centroid |
| SUDV_056 | SUDV | 2011 | Luweero | 32.49944 | 0.83306 | district centroid |
| SUDV_057 | SUDV | 2011 | Masaka | 31.73611 | –0.34111 | district centroid |
| Bundibugyo ebolavirus (BDBV) – 2007 outbreak |  |  |  |  |  |  |
| BDBV_001 | BDBV | 2007 | Ntoroko | 30.40000 | 1.10000 | district centroid |
| BDBV_002 | BDBV | 2007 | Bundibugyo | 30.06700 | 0.71700 | district centroid |
| BDBV_003 | BDBV | 2007 | Kabarole | 30.30000 | 0.60000 | district centroid |
| BDBV_004 | BDBV | 2007 | Kyenjojo | 30.61700 | 0.61700 | district centroid |
| BDBV_005 | BDBV | 2007 | Kikuube | 31.13300 | 1.21700 | district centroid |
| BDBV_006 | BDBV | 2007 | Kanungu | 29.78300 | –0.95000 | district centroid |
| BDBV_007 | BDBV | 2007 | Rukungiri | 29.93300 | –0.78300 | district centroid |
| Zaire ebolavirus (EBOV) – imported cases |  |  |  |  |  |  |
| EBOV_001 | EBOV | 2018* | Kisoro | 29.68300 | –1.28300 | district centroid |
| EBOV_002 | EBOV | 2018* | Kanungu | 29.78300 | –0.95000 | district centroid |
| EBOV_003 | EBOV | 2018* | Kasese | 30.08300 | 0.18300 | district centroid |
| EBOV_004 | EBOV | 2018* | Bundibugyo | 30.06700 | 0.71700 | district centroid |
| EBOV_005 | EBOV | 2018* | Ntoroko | 30.40000 | 1.10000 | district centroid |
| EBOV_006 | EBOV | 2018* | Kabaale | 31.25000 | 2.45000 | district centroid |
| EBOV_007 | EBOV | 2018* | Kasese | 31.16700 | 3.00000 | district centroid |

## **Table S4. Tuning grid results from ENMeval (AICc and metrics for each configuration).**

| **Model** | **Feature classes** | **Beta multiplier** | **AICc** | **Delta AICc** | **wAICc** | **Mean test AUC** | **Mean test Boyce** | **Mean test OR10** | **No parameters** |
| --- | --- | --- | --- | --- | --- | --- | --- | --- | --- |
| Pooled | LQHP | 1.5 | 1980.0 | 0.0 | 0.2080 | 0.850 | 0.600 | 0.095 | 26 |
| Pooled | LQHP | 1.0 | 1980.5 | 0.5 | 0.1620 | 0.848 | 0.593 | 0.098 | 27 |
| Pooled | LQHP | 2.0 | 1980.5 | 0.5 | 0.1620 | 0.848 | 0.593 | 0.098 | 25 |
| Pooled | LQH | 1.5 | 1982.0 | 2.0 | 0.0770 | 0.845 | 0.580 | 0.101 | 22 |
| Pooled | LQHP | 0.5 | 1982.0 | 2.0 | 0.0770 | 0.840 | 0.570 | 0.105 | 28 |
| Pooled | LQHP | 2.5 | 1982.0 | 2.0 | 0.0770 | 0.840 | 0.570 | 0.105 | 24 |
| Pooled | LQH | 1.0 | 1982.8 | 2.8 | 0.0530 | 0.843 | 0.573 | 0.103 | 23 |
| Pooled | LQH | 2.0 | 1982.8 | 2.8 | 0.0530 | 0.843 | 0.573 | 0.103 | 21 |
| Pooled | LQ | 1.5 | 1984.0 | 4.0 | 0.0280 | 0.840 | 0.550 | 0.107 | 16 |
| Pooled | LQHP | 3.0 | 1984.5 | 4.5 | 0.0220 | 0.828 | 0.533 | 0.117 | 23 |
| Pooled | LQ | 1.0 | 1985.0 | 5.0 | 0.0170 | 0.838 | 0.543 | 0.110 | 17 |
| Pooled | LQ | 2.0 | 1985.0 | 5.0 | 0.0170 | 0.838 | 0.543 | 0.110 | 15 |
| Pooled | LQH | 0.5 | 1985.0 | 5.0 | 0.0170 | 0.835 | 0.550 | 0.111 | 24 |
| Pooled | LQH | 2.5 | 1985.0 | 5.0 | 0.0170 | 0.835 | 0.550 | 0.111 | 20 |
| Pooled | LQ | 0.5 | 1988.0 | 8.0 | 0.0040 | 0.830 | 0.520 | 0.117 | 18 |
| Pooled | LQ | 2.5 | 1988.0 | 8.0 | 0.0040 | 0.830 | 0.520 | 0.117 | 14 |
| Pooled | LQH | 3.0 | 1988.8 | 8.8 | 0.0027 | 0.818 | 0.503 | 0.127 | 19 |
| Pooled | LQ | 3.0 | 1992.0 | 12.0 | 0.0005 | 0.808 | 0.470 | 0.147 | 13 |
| Pooled | L | 2.0 | 1993.0 | 13.0 | 0.0003 | 0.830 | 0.500 | 0.127 | 5 |
| Pooled | L | 1.5 | 1994.0 | 14.0 | 0.0002 | 0.838 | 0.513 | 0.117 | 6 |
| Pooled | L | 2.5 | 1994.0 | 14.0 | 0.0002 | 0.818 | 0.483 | 0.137 | 4 |
| Pooled | L | 1.0 | 1997.0 | 17.0 | 0.00005 | 0.828 | 0.480 | 0.137 | 7 |
| Pooled | L | 3.0 | 1997.0 | 17.0 | 0.00005 | 0.798 | 0.440 | 0.167 | 3 |
| Pooled | L | 0.5 | 2002.0 | 22.0 | 0.000004 | 0.830 | 0.450 | 0.157 | 8 |
| Pooled | H | 2.0 | 2004.0 | 24.0 | 0.000001 | 0.815 | 0.485 | 0.132 | 13 |
| Pooled | H | 1.5 | 2005.2 | 25.2 | 0.0000007 | 0.823 | 0.498 | 0.122 | 14 |
| Pooled | H | 2.5 | 2005.2 | 25.2 | 0.0000007 | 0.803 | 0.468 | 0.142 | 12 |
| Pooled | H | 1.0 | 2009.0 | 29.0 | 0.0000001 | 0.813 | 0.465 | 0.152 | 15 |
| Pooled | H | 3.0 | 2009.0 | 29.0 | 0.0000001 | 0.783 | 0.425 | 0.182 | 11 |
| Pooled | H | 0.5 | 2017.2 | 37.2 | 0.00000001 | 0.810 | 0.420 | 0.167 | 16 |

## **Table.S5. Fold-wise performance metrics for pooled model.**

| **Fold** | **Train AUC** | **Test AUC** | **Test Boyce** | **Train OR10** | **Test OR10** | **Threshold 10pctTP_cloglog** | **Suitable area_percent** |
| --- | --- | --- | --- | --- | --- | --- | --- |
| Fold 1 | 0.924 | 0.840 | 0.55 | 0.095 | 0.092 | 0.081 | 24.6 |
| Fold 2 | 0.935 | 0.862 | 0.63 | 0.101 | 0.105 | 0.086 | 26.1 |
| Fold 3 | 0.929 | 0.853 | 0.60 | 0.099 | 0.098 | 0.084 | 25.4 |
| Fold 4 | 0.921 | 0.825 | 0.52 | 0.101 | 0.109 | 0.088 | 26.8 |
| Mean | 0.927 | 0.845 | 0.58 | 0.099 | 0.101 | 0.085 | 25.7 |
| SD | 0.006 | 0.016 | 0.05 | 0.003 | 0.008 | 0.003 | 0.9 |

# **Supplementary Data and Code Notes**

The complete workflow, georeferenced spill-over dataset (with spatial unit (villages/parishes/sub counties) centroids jittered by ±2 km to protect privacy), final 1-km GeoTIFF risk rasters, and R markdown notebooks are archived on Zenodo (<https://doi.org/10.5281/zenodo.15600735>) and mirrored on GitHub (<https://github.com/gpaasi/ebola-MaxEnt-ecological-niche-modeling-uganda>).

**References**

1.	Fick, S.E. and R.J. Hijmans, *WorldClim 2: new 1-km spatial resolution climate surfaces for global land areas.* International Journal of Climatology, 2017. **37**(12): p. 4302-4315.

2.	*Shuttle Radar Topography Mission (SRTM) | NASA Earthdata*.

3.	*WorldPop :: Population Density*.

4.	Jagadesh, S., et al., *Mapping Global Bushmeat Activities to Improve Zoonotic Spillover Surveillance by Using Geospatial Modeling.* Emerg Infect Dis, 2023. **29**(4): p. 742-750.

5.	Guo, K., Q. Ji, and D. Zhang, *Climate Physical Risk Index (CPRI)*. 2024, figshare.

6.	User, G.B.O., *Occurrence Download*. 2024, The Global Biodiversity Information Facility.
