# Supplementary material for: High‐Resolution Mapping of Environmental Suitability for Reported Ebola Spillover Events in Uganda to Inform Surveillance and Preparedness
Source: J Trop Med. 2026 Aug 16;2026:9889973. doi: 10.1155/jotm/9889973 (PMC13478722; doi:10.1155/jotm/9889973)
Supplement: Supplementary file 2 [file JOTM-2026-9889973-s002.docx]

ISLE-ReSt checklist [1] for. High-resolution ecological niche maps identify Ebola spillover risk hotspots in Uganda for targeted district surveillance and preparedness.

| **No.** | **Section** | **Recommendation** | **Status** | **Where in manuscript** | **Section** | **Rationale** |
| --- | --- | --- | --- | --- | --- | --- |
| 1 | Title | Title states study design, primary exposure, and primary outcome | Done | Title page | Title | States ecological niche maps (design), Ebola spillover suitability (exposure framing), and targeted surveillance/preparedness hotspots (outcome use). |
| 2 | Abstract | Design, setting, exposure sources, outcomes, key results with precision, conclusions | Done | Abstract | Abstract | Summarizes setting (Uganda), occurrence definition and window, modelling/tuning and spatial CV, operational threshold, tiering and key performance metrics with ranges. |
| 3 | Introduction | Background and conceptual framework linking environment and health | Done | Introduction opening paragraphs | Introduction | Links zoonotic spillover and reservoir ecology with landscape drivers, and motivates district-scale risk mapping. |
| 4 | Introduction | Specific objectives and any prespecified hypotheses | Done | Introduction final paragraph | Introduction | Explicitly states aims to generate species-resolved 1 km surfaces, quantify predictor contributions, and translate suitability into hotspots. |
| 5 | Methods | Study design and setting, locations, calendar period, spatial scale of exposure measurement | Done | Study area; Occurrence data and georeferencing | Materials and Methods | Defines Uganda as the analysis extent and describes the event window and locality approach for spillover mapping. |
| 6 | Methods | Participants, eligibility, selection, recruitment, retention | N/A | Not applicable (secondary ecological modelling) | Declarations and Methods | No recruited participants, analyses use outbreak localities and environmental rasters. |
| 7 | Methods | Sample size rationale or calculation, attrition handling | N/A | Not applicable | Abstract; Methods | No participant sample size, modelling basis is occurrence locality count plus background points. |
| 8 | Methods | Data linkage and geocoding method, reference data, CRS, software, percent geocoded | Done | Occurrence data and georeferencing; Environmental predictors and data processing | Materials and Methods | Describes georeferencing strategy, coordinate precision categorisation, thinning, harmonisation of rasters, and projected CRS (EPSG:32636). |
| 9 | Methods | Variables defined and measured for outcomes, exposures, predictors, confounders, effect modifiers | Done | Definition of spillover locality; Environmental predictors; Collinearity screening | Materials and Methods | Defines the occurrence outcome proxy (spillover locality) and specifies candidate predictors and retained predictor set after collinearity screening. |
| 10 | Methods | Bias considered and addressed, including selection, information, spatial biases | Done | Background sampling and correction for uneven detection effort | Materials and Methods | Includes bias-informed background sampling using a health-facility density bias surface to reduce reporting opportunity bias. |
| 11 | Methods | Quantitative variables, handling, transformations, groupings | Done | Collinearity screening; Suitability thresholds and risk stratification | Materials and Methods | States correlation cutoff |
| 12 | Methods | Spatial data sources, collection time, spatial and temporal resolution, preprocessing | Done | Environmental predictors and data processing; Table 1 | Materials and Methods | Lists major spatial datasets (WorldClim, SRTM, WorldPop, GBIF, ClimAfr), and describes harmonising to 1 km resolution and clipping to Uganda. |
| 13 | Methods | Remote sensing sensor or product details | Done | Environmental predictors and data processing | Materials and Methods | Uses remotely sensed or derived environmental products (WorldClim bioclim, SRTM terrain) as predictors. |
| 14 | Methods | GPS and accelerometry device info | N/A | Not applicable | Not applicable | No participant device-based data collection is used in the study. |
| 15 | Methods | Smartphone apps or other sensors | N/A | Not applicable | Not applicable | No app-based sensor streams are used. |
| 16 | Methods | Spatial exposure construction, buffers, network versus Euclidean distance, software | Done | Suitability thresholds and risk stratification; Spatial processing notes | Materials and Methods | Explains how the continuous surface is thresholded and stratified into tiers for operational use, including resampling for cartographic output. |
| 17 | Methods | Matching spatial and health data, time windows, exposure lags, activity space, aggregation | Done | Occurrence definition, georeferencing, event date recording | Materials and Methods | Describes linkage of outbreak events to spatial predictors through georeferenced spillover localities and event dates. |
| 18 | Methods | Statistical methods, clustering, spatial autocorrelation, residuals, sensitivity, missing data | Done | Model fitting, tuning, evaluation; Spatial cross-validation; Sensitivity analyses | Materials and Methods | Describes ENMeval tuning, four-fold spatial block cross-validation, metrics (AUC, Boyce, OR10), and checkerboard2 sensitivity. |
| 19 | Results | Participant flow and numbers at each stage, geocoding success | N/A | Not applicable | Not applicable | No participant flow applies to an ecological niche model using event localities. |
| 20 | Results | Descriptive data, exposure distributions, follow up time | Done | Results sections describing suitability mask and tier exposure | Results | Reports suitable area fraction and population distribution across tiers, and describes geographic clustering of high-tier suitability. |
| 21 | Results | Main results with estimates and precision, adjusted confounders, spatial diagnostics | Done | Results summary in Abstract and Results narrative | Abstract; Results | Provides held-out performance ranges (AUC, Boyce, OR10), operational threshold, suitable area %, and tier 1 land and population share. |
| 22 | Discussion | Key results in relation to objectives and existing evidence | Done | Discussion opening and interpretation paragraphs | Discussion | Interprets hotspot belts and virus-specific patterns, relating them to surveillance and preparedness targeting. |
| 23 | Discussion | Limitations including temporal mismatch, misclassification, nonstationarity, mobility, migration | Done | Discussion limitations paragraphs | Discussion | Discusses detection/reporting bias risk and interpretation limits of presence-only suitability (and addresses mitigation steps). |
| 24 | Discussion | Generalizability across places and times | Done | Discussion and Conclusion framing | Discussion; Conclusion | Frames outputs as an operational prioritisation framework (tiers and hotspot belts) that can guide district preparedness and One Health surveillance. |
| 25 | Other | Funding sources and roles | Done | Declarations | Declarations | Funding is stated as none. |
| 26 | Other | Data and code sharing, conflicts of interest, access terms, code location | Done | Availability of data and materials; Competing interests | Declarations | Provides repository locations (Zenodo and GitHub) and declares no competing interests.  The complete workflow, georeferenced spill-over dataset (with spatial unit (villages/parishes/sub counties) centroids jittered by ±2 km to protect privacy), final 1-km GeoTIsFF risk rasters, and R markdown notebooks are archived on Zenodo (<https://doi.org/10.5281/zenodo.15600735>) and mirrored on GitHub (<https://github.com/gpaasi/ebola-MaxEnt-ecological-niche-modeling-uganda>). |

1.	Jia, P., et al., Spatial Lifecourse Epidemiology Reporting Standards (ISLE-ReSt) statement. Health & Place, 2020.

61: p. 102243.
